# Diffuse Leiomyomatosis of the Uterus: A Diagnostic Enigma

**DOI:** 10.7759/cureus.29595

**Published:** 2022-09-26

**Authors:** Indira Prasad, Sudwita Sinha, Upasna Sinha, Tarun Kumar, Jyoti Singh

**Affiliations:** 1 Department of Obstetrics and Gynecology, All India Institute of Medical Sciences, Patna, IND; 2 Department of Radiology, All India Institute of Medical Sciences, Patna, IND; 3 Department of Pathology/Lab Medicine, All India Institute of Medical Sciences, Patna, IND

**Keywords:** surgical management, uterine leiomyomatosis, dul, diffuse uterine leiomyomatosis, leiomyomatosis

## Abstract

We present a case of diffuse uterine leiomyomatosis in a 38-year-old nulliparous female presenting with abdominal distension and infertility, which is very rarely reported and commonly misdiagnosed. Magnetic resonance imaging (MRI) of the abdomen and pelvis showed an enlarged uterus of size 25 × 20 ×13 cm with a few fibroids in the lower uterine segment and pressure effects on the ureter, causing hydroureteronephrosis. The fundal region and upper uterine segment were extensively thickened with a mildly thinned-out junctional zone. A total abdominal hysterectomy was performed, and the diagnosis of diffuse leiomyomatosis of the uterus was confirmed on histopathological examination. The post-operative period was complicated by hypovolemic shock, which was managed by transfusion of multiple units of blood, blood components, and hemostatics. On the ninth post-operative day, the patient gained full recovery and was discharged.

## Introduction

Leiomyomas are common tumors of the uterus that present radiologically as circumscribed masses [1]. There are also unusual patterns of leiomyoma growth described in the literature, with diffuse leiomyomatosis of the uterus being one of them [1]. Diffuse uterine leiomyomatosis (DUL) is a benign rare condition in which the uterus is symmetrically enlarged due to almost complete replacement of the myometrium by innumerable poorly defined, confluent nodules [2]. Till date, less than 50 sporadic cases have been reported in literature. The first reported case described by Murray and Glynn in 1924 as “complete uterine fibromyomatosis” was subsequently renamed “diffuse uterine leiomyomatosis” by Lapan and Solomon in 1979 [3]. Its etiology is still not completely understood [2]. A clonality analysis study of several lesions from a patient had shown that all lesions had a non-random X-chromosome inactivation pattern, consistent with a monoclonal neoplastic population of cells [4]. However, different X chromosomes were inactivated in different foci of the tumor, supporting the independent origin of neoplastic clones and rejecting the possibility of a single clonal origin of all tumor cells [4]. These results suggest that DUL may be an exuberant example of uniform and diffuse involvement of the entire myometrium by multiple leiomyomata [4]. Microscopically, it is characterized by typical, spindle-shaped smooth muscle cells forming an interlacing fascicular arrangement with ill-defined cell borders, eosinophilic filamentous cytoplasm, cigar-shaped nuclei, small nucleoli, and uniformly low mitotic activity [3]. Initial presenting symptoms include abdominal pain, abnormal uterine bleeding, menorrhagia, dysmenorrhea, pelvic pressure, and infertility [2]. Hormones usually fail to control the symptoms, anemia, or tumor growth once the treatment is stopped [1,2], that is, as long as hormonal therapy is continued, patients may have slight control of symptoms, but the symptoms recur as soon as treatment is discontinued. So far in terms of treatment, hysterectomy is the only permanent solution for these patients [1,2]. However, the problem arises as a majority of these patients are in their third to fourth decade of life and may desire future pregnancy [1]. Successful pregnancies have been reported in women treated with gonadotropin-releasing hormone agonists or minimally invasive hysteroscopic myomectomy by removing submucosal leiomyomas [5]. Other treatment options include uterine artery embolization, high-intensity focused ultrasound (HIFU), and sirolimus [5]. Two cases of recurrence after conservative surgery have been reported [5].

## Case presentation

A 38-year-old nulliparous female with diffuse leiomyomatosis of the uterus presented to the Gynecology OPD with complaints of lump abdomen and increasing abdominal distension for the past one year. She noticed the lump-like appearance of her abdomen a year ago, for which she consulted a local clinician who advised her to get an ultrasonography done, which showed swelling of the uterus. Besides the lump-like abdomen, the patient did not have any other symptoms that were of concern to her and chose not to follow up with her physician till the uterus reached an alarming size. She also complained of not being able to conceive for the last 10 years despite having regular unprotected intercourse with her partner. However, she had also not done any workup for her subfertility, assuming her lump-like abdomen to be the reason behind it. She did not provide any history of heavy menstrual bleeding, dysmenorrhea, dyspareunia, or abdominal pain, nor did she give any history of nausea, vomiting, anorexia, indigestion, dyspepsia, or bloating sensation. It was not until a few days back when she started having slight respiratory discomfort occasionally due to her huge abdominal distension that she thought of getting a consultation. She attained her menarche at the age of 13 years and had been having normal, regular menstrual cycles since then of three to four days with a cycle length of approximately 30 days. There was no associated heavy flow, passage of clots, dysmenorrhea, or dyspareunia. There was no significant past medical or surgical history, no history of allergies, and no history of drug use or contraceptive use. On examination, she was found to have a cachectic look. Her body weight was 54 kg and her height was 153 cm. She was afebrile with a pulse rate of 90 beats per minute, respiratory rate of 20 breaths per minute, and blood pressure of 100/60 mm Hg. Mild pallor was observed. However, there was no icterus, cyanosis, clubbing, leg varicosities, or edema. No prominent neck veins and no enlarged neck nodes or supraclavicular nodes were palpable. A systemic examination did not reveal any abnormality. Her breast examination was normal. On inspection, the abdomen was hugely distended with flanks full, everted umbilicus, and prominent umbilical veins. No previous scar was seen. On palpation, a firm mass was palpable from the pubic symphysis up to the xiphisternum, occupying the whole of abdomen with a smooth surface, regular margins, and slightly restricted side-to-side mobility, probably due to its huge size. The lower margin of the mass could not be reached. Per speculum examination, findings were normal except for a pulled-up cervix. On bimanual examination, all fornices were free, no nodules were palpable in the pouch of Douglas, and the uterus was not appreciated. No abnormal findings were noticed on the rectal examination either.

**Figure 1:**
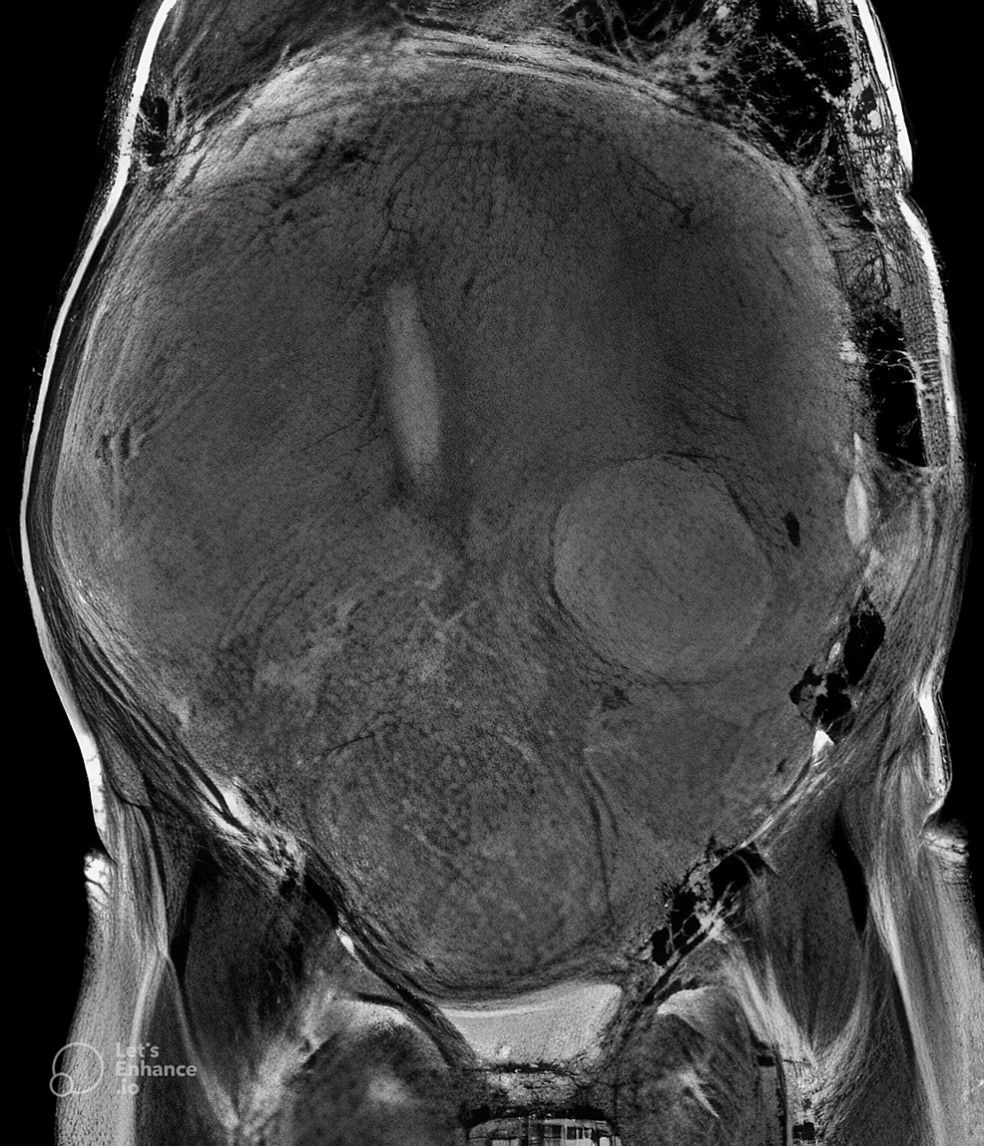
Coronal T2-weighted imaging showing a grossly enlarged uterus with multiple T2 heterogenous signal intensity intramural fibroids in the lower uterine segment and extensively thickened myometrium in the fundal region and upper uterine segment (1.5 Tesla MRI).

**Figure 2:**
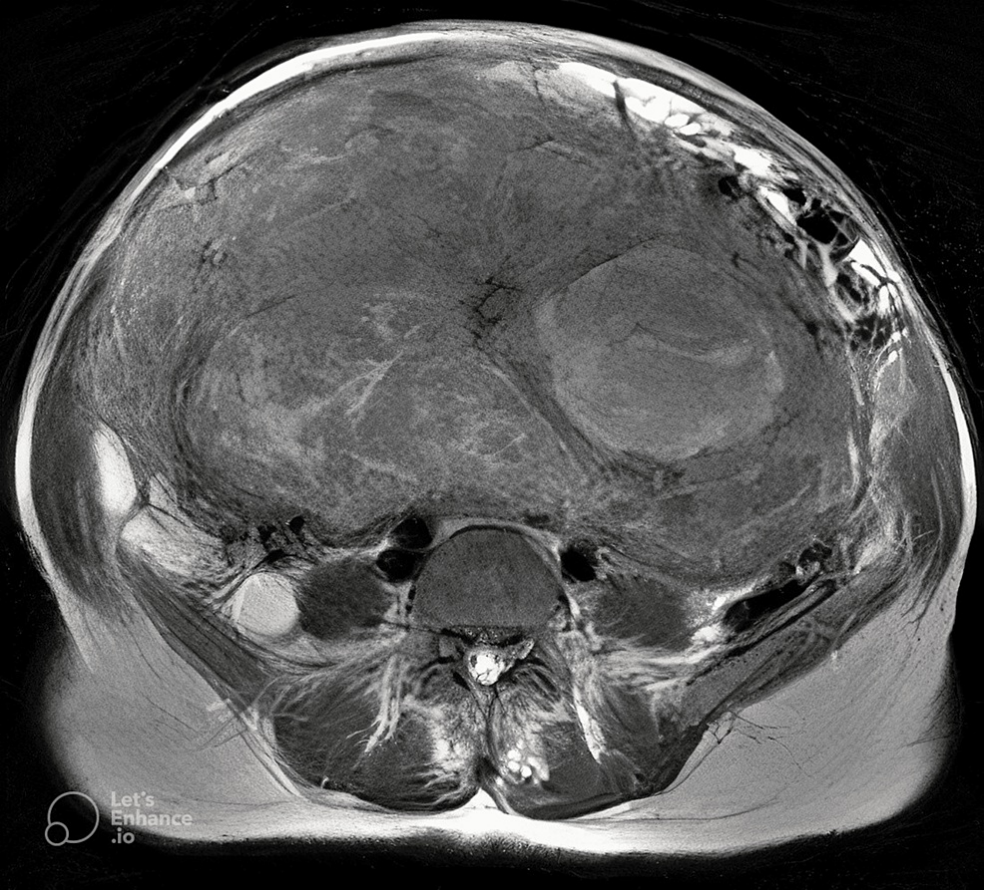
Axial T2-weighted image showing a grossly enlarged uterus with multiple T2 heterogenous signal intensity intramural fibroids in the lower uterine segment and extensively thickened myometrium in the fundal region and upper uterine segment (1.5 Tesla MRI).

To our surprise, the transabdominal ultrasound revealed a large adenomytoic uterus, as we would expect an ovarian neoplasm in such a scenario. Using a 3T MRI scanner on a multichannel body coil system, the uterus was found to be grossly enlarged, measuring about 13 x 20 x 25 cm, which extended superiorly in the abdomen, abutting the inferior surface of the liver. Multiple well-defined, T2 heterogenous signal intensity intramural fibroids were seen in the lower uterine segment, with the largest measuring about 15 x 11 cm. The fundal and upper uterine segments were extensively thickened with a mildly thinned-out junctional zone. The enlarged uterus was compressing the distal ureter, leading to bilateral moderate hydroureteronephrosis. A few tortuous collaterals were noted in the pelvis and omentum. The endometrium measured 8.5 mm with thinning of the endometrium-myometrium junctional zone in the upper uterine segment. Both ovaries were not seen separately from the enlarged uterus. A differential diagnosis of myometrial myxoidosis was considered apart from the possibility of diffuse leiomyomatosis of the uterus. Figures 1, 2 show the images seen on MRI.

The patient was told in detail about the condition, and considering her nulliparous state, management options were discussed with her. She opted for surgery. Myomectomy could not have been a viable management option as it would not rid the patient totally of her abdominal mass; hence, after explaining the need for surrogacy and adoption for future fertility and taking her informed consent, the decision to perform a total abdominal hysterectomy was taken. A pre-anesthetic check-up was done and pre-operative investigations were sent, all of which were within normal limits except for a decreased hemoglobin count of 8.5 g/dL.

**Figure 3:**
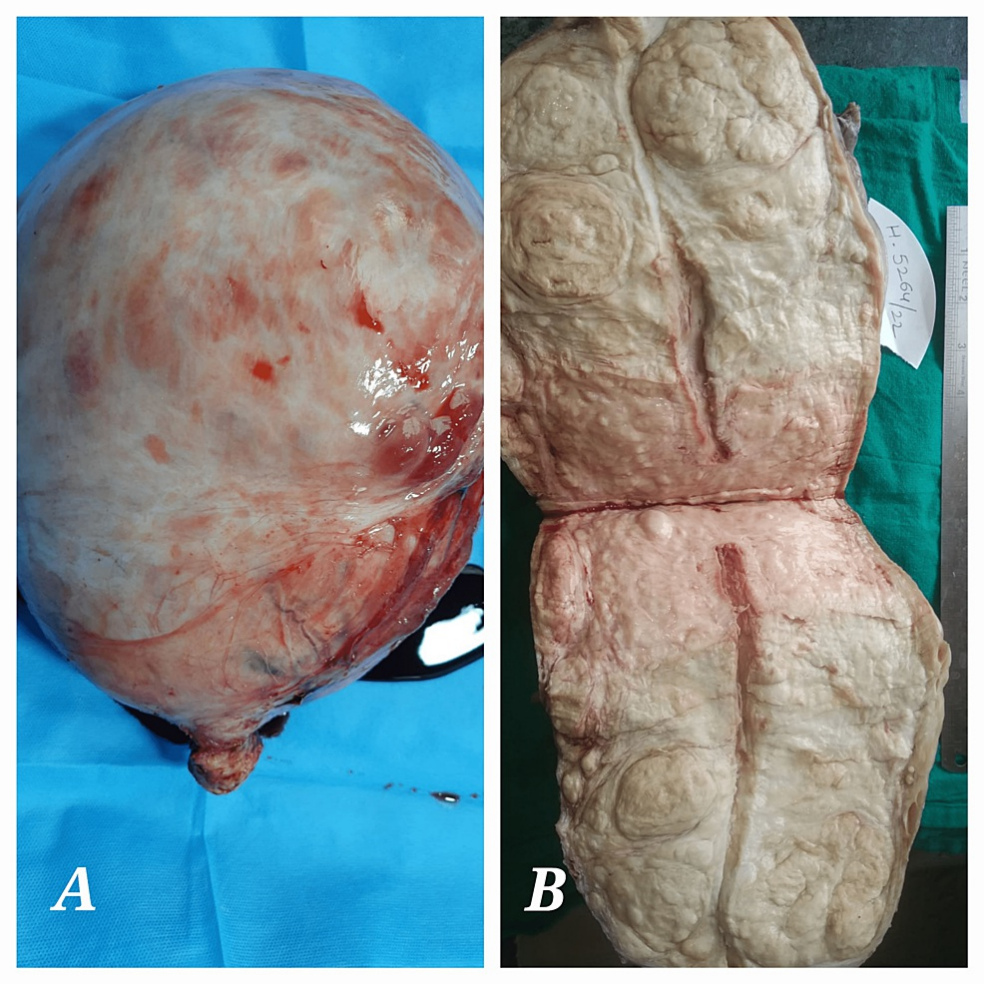
Image showing (A) gross appearance and (B) cut section of the uterus.

**Figure 4:**
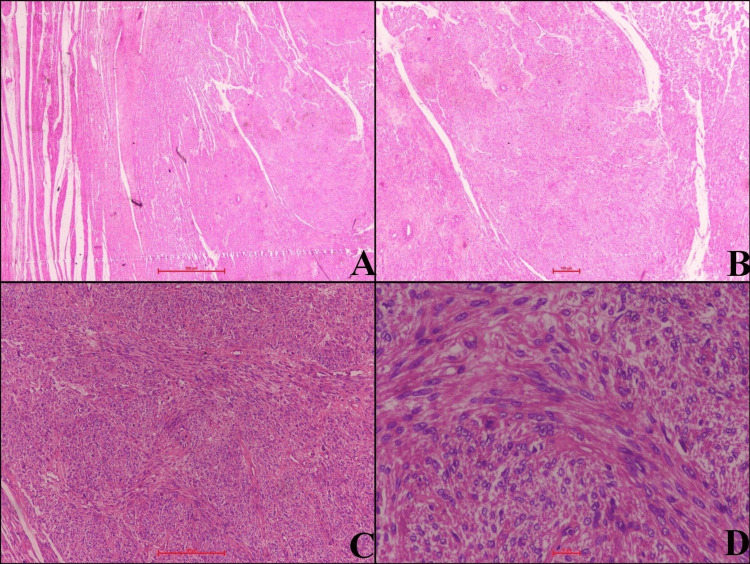
Image showing slides on histopathological examination. (A) The section shows a poorly circumscribed uterine smooth muscle tumor (H&E x2). (B) Benign uterine smooth muscle tumor with the presence of large thick blood vessels (H&E x4). (C) Tumor with intersecting fascicles (H&E x10). (D) Tumor with oval to spindle cells having vesicular to hyperchromatic nuclei, minimal pleomorphism, and scant-to-moderate eosinophilic cytoplasm. No atypical mitosis was seen (H&E x40).

After taking the patient’s informed consent and arranging adequate blood products, a total abdominal hysterectomy was performed under all antiseptic precautions. Epidural anesthesia combined with general anesthesia was administered. The patient was laid down in a supine position, following which antiseptic painting and draping and bladder catheterization were performed. The abdominal cavity was opened by a midline vertical incision extending above the umbilicus. A giant-sized uterus weighing 5,600 grams was delivered. The right tube and ovary were not seen, and the left tube and ovary were normal. A total abdominal hysterectomy was performed, and the specimen was sent for histopathological examination. Hemostasis was achieved. The intraperitoneal drain was kept in situ, and the abdomen was closed in layers after counting mops, needles, and instruments. The total operating time was 130 minutes, and the estimated blood loss was 800 mL. There was intra-operative fall of blood pressure, which was managed by fluid resuscitation, blood transfusion, and vasopressors. The patient was monitored in the ICU post-operatively for four consecutive days and was shifted to the ward on the fifth day after being stabilized. Intra-operatively and post-operatively, the patient received a total of six packed red blood cell units, 13 fresh frozen plasma units, four platelet concentrate units, and two cryoprecipitate units as required over a period of four days as the patient developed coagulopathy and hemorrhagic shock in the immediate post-operative period. The drain output for the first two days was around 1,000 mL per 24 hours, which was serosanguinous, and subsequently started decreasing, and drain was removed on the seventh post-operative day. Post-operatively, the patient was managed with IV antibiotics, antacids, analgesics, deep vein thrombosis prophylaxis, chest physiotherapy, and incentive spirometry. The catheter was removed on the fifth post-operative day. The patient was gradually started on oral sips, liquid diet, and soft diet, and was on a normal diet by the sixth post-operative day. The patient was discharged on the ninth post-operative day after the total removal of skin stitches. The histopathology report confirmed the diagnosis of diffuse leiomyomatosis of the uterus as mentioned in the MRI. Figures 3, 4 show the images on gross examination, cut section, and histopathologic examination.

## Discussion

DUL is a benign neoplasm of smooth muscle of the uterus with unknown etiology [3]. Knowledge regarding this disease is limited due to its rare reporting. [3]. Early diagnosis is difficult, and it is often misdiagnosed before surgery due to the lack of specific manifestations [3]. Its diagnosis is largely dependent on pre-operative MRI and post-operative histopathology [3]. Currently, there is no unified diagnostic method for the same [3]. MRI aids in early identification and proper pre-operative evaluation [3].

Although ultrasonography should be the first line of imaging modality to assess the pelvic cavity and uterine or ovarian disorders, MRI is the optimal imaging modality frequently used due to its multiplanar imaging capabilities, excellent soft tissue contrast, and complementary role in pre-treatment mapping [1]. On imaging, diffuse leiomyomatosis was found to have indistinct borders and coalesce together in contrast to typical leiomyomas, which are well-circumscribed masses with an asymmetrical involvement of the uterus [1]. On gross examination, DUL, which is a type of myometrial hypertrophy, presents as thick and irregular myometrium resembling adenomyosis, with numerous small myomas. On microscopic examination, the cells are found to be typical, uniform, bland spindled smooth muscle cells grouped in small leiomyomas that are less circumscribed than typical leiomyomas [6].

Differential diagnoses include diffuse uterine adenomyosis, diffuse endometrial hypertrophy, multiple leiomyomas, disseminated peritoneal leiomyomatosis, uterine lymphangiomyomatosis, intravascular leiomyomatosis, hereditary leiomyomatosis and renal cell carcinoma (HLRCC), Alport syndrome with diffuse leiomyomatosis (ASDL), and endometrial stromal sarcoma [3]. Diffuse uterine adenomyosis and diffuse endometrial hypertrophy can be excluded by histopathological examination. DUL can be distinguished from multiple leiomyomas by the uniform symmetrical involvement of the entire myometrium by smooth muscle nodules with indistinct borders, whereas multiple leiomyomas have asymmetrical uterine involvement with sharp circumscribed masses [7]. Disseminated peritoneal leiomyomatosis is a benign entity where the presence of uterine leiomyomas is associated with multiple mature smooth muscle nodules affecting the mesentery, visceral, and parietal fascia of the peritoneum [8]. Uterine lymphangiomatosis is characterized by the invasion of pulmonary vascular spaces [8]. Intravascular leiomyomatosis has a creamy to yellow color, with worm-like intravascular extensions of smooth muscle tumor having multinodular irregular or indistinct margins and the presence of some or all of the neoplastic smooth muscle within the vascular channels on histopathology [7]. HLRCC is an autosomal dominant disorder caused by mutations of the fumarate hydratase (FH) gene, characterized by cutaneous leiomyoma, and concomitant with multiple uterine leiomyomas and renal cell carcinoma [3]. ASDL is an X-linked inherited disorder resulting from mutations in the COL4A5 gene where myoma lesions are widely spread over the esophagus, trachea, bronchi, and genitalia [3]. Endometrial stromal sarcoma is characterized by invasive growth having an abrupt transition with the normal myometrium and a sheetlike growth pattern, and small neoplastic cells with round to oblong nuclei [7].

For women desiring fertility, conservative treatments should be opted for, which might include medical management with hormones such as gonadotrophin-releasing hormone agonists or conservative surgeries [3]. Conservative surgeries may include novel myomectomy techniques involving a longitudinal dissection of the uterus in the midline, hysteroscopic surgery using cold graspers combined with an electric loop, and uterine artery embolization [9-12]. However, a risk of recurrence after myomectomy has been reported [5]. Hence, the only curative therapy as of now is hysterectomy [3].

Till date, less than 50 such cases have been reported in literature. Successful pregnancy outcomes after conservative surgeries have also been reported. Pai et al. reported DUL in a 16-year-old girl [13]. Thomas et al. reported DUL with uterine rupture and benign metastatic lesions of the bone in a 35-year-old nulliparous woman with fetal demise [14]. Robles-Frias et al. reported DUL with ovarian and parametrial involvement [8]. Agarwal et al. reported a case of diffuse leiomyomatosis of the uterus diagnosed during pregnancy with successful vaginal delivery [15]. Hassan et al. reported a case of primigravida with DUL and IUGR, necessitating a cesarean section and hysterectomy [16].

## Conclusions

DUL is a rarely reported benign neoplasm of the uterus whose etiology is not yet known. It is very commonly misdiagnosed, and, in most cases, diagnosis is confirmed only after a histopathological examination of the hysterectomy specimen. An MRI of the pelvis is of great help in diagnosis and pre-operative evaluation. Hormonal treatments are usually non-responsive in the long term, and recurrence after conservative surgery has been reported. Owing to this, the last resort for complete cure at present is hysterectomy, even in younger patients in the third and fourth decades of life.
